# Dr. Wallace’s Note on an Antidote to Corrosive Sublimate

**Published:** 1842-10-08

**Authors:** J. M. Wallace

**Affiliations:** Philadelphia


### MEDICAL EXAMINER.

NEW SERIES.

No. 41.] PHILADELPHIA, OCTOBER 8, 1842. [Vol. I.

### ANTIDOTE TO CORROSIVE SUBLIMATE.

To the Editors of the Medical Examiner.

Gentlemen,—The October number of the American Journal of the Medical Sciences contains, under the head of Progress of the Medical Sciences, the following:—

"*Antidote to Corrosive Sublimate.*—M. Mialhe, in a note read to the Academy of Paris, August 16, states, as the results from his experiments, that the hydrated proto-*sulphate* of iron (a substance quite innocuous,) possesses the property of instantly decomposing corrosive sublimate. The products of the decomposition are the proto-chloride of iron, and the *bi-sulphate* of mercury, inert substances." Page 496.

There is obviously an error here. The only precipitate procured by this preparation of iron is the peroxide. The translator has no doubt mistaken proto-*sulphate* for proto-*sulphuret*, and *bisulphate* for *bisulphuret*. The former substance (proto-sulphate) has no effect in decomposing corrosive sublimate, but the latter (proto-sulphuret) *is* an antidote and acts by double decomposition. The mode of preparing this new antidote may not be without interest.

Add a solution of sulphuret of potassium (Hepar sulphuris) to a solution of proto-sulphate of iron, (copperas,) and the black proto-sulphuret of iron is precipitated. Wash this with water, and you have the antidote ready for use. When this is added to a solution of corrosive sublimate, the reaction is such, that two equivalents of proto-sulphuret of iron, and one equivalent of bichloride of mercury, yield two equivalents of the protochloride of iron, and one equivalent of the bisulphuret of mercury, or vermilion, and not the slightest trace of mercury is found in the filtered liquid. We have thus an antidote for corrosive sublimate, as efficient as the hydrated sesquioxide of iron for arsenic.

While on this subject, I may mention that I have just witnessed another proof of the efficacy of the latter article. A child, 18 months old, ate some bread and butter, on which arsenic had been thickly spread for rats; he was at a distance from the city, and the iron was not procured for two hours after the poison had been taken. He is now recovering without a bad symptom. In this case the freshly prepared article was procured by Mr. Procter, in *eight minutes* from the time he received the order, by precipitating from a solution of the persulphate of iron by ammonia, as recommended by him in his paper published in the Journal of the College of Pharmacy, and in the Medical Examiner, for this year, No. 19, p. 295.

I am, gentlemen,

Your obedient servant,

J. M. Wallace.

*Philadelphia*, October 6th, 1842.

